# Ameloblastoma RNA profiling uncovers a distinct non-coding RNA signature

**DOI:** 10.18632/oncotarget.13889

**Published:** 2016-12-10

**Authors:** Haleh Davanian, Anangi Balasiddaiah, Robert Heymann, Magnus Sundström, Poppy Redenström, Mikael Silfverberg, David Brodin, Matti Sällberg, Sven Lindskog, Carina Kruger Weiner, Margaret Chen

**Affiliations:** ^1^ Department of Dental Medicine, Karolinska Institutet, Huddinge, Sweden; ^2^ Department of Laboratory Medicine, Karolinska Institutet, Huddinge, Sweden; ^3^ Rudbecklaboratory, Molecular Pathology Unit, Department of Pathology, Uppsala University Hospital and Department of Immunology, Genetics and Pathology, Uppsala University, Sweden; ^4^ Department of Biosciences and Nutrition, Bioinformatics and Expression Analysis SciLifeLab, Karolinska Institutet, Huddinge, Sweden; ^5^ Department of Oncology and Pathology, Karolinska Institutet, Huddinge, Sweden; ^6^ Clinical Pathology and Cytology, Karolinska University Hospital, Karolinska University Hospital, Solna, Sweden; ^7^ The Clinic of Oral and Maxillofacial Surgery, Karolinska University Hospital, Huddinge, Sweden

**Keywords:** ameloblastoma, biomarkers, transcriptome, ncRNA, gene expression analysis

## Abstract

Ameloblastoma of the jaws remains the top difficult to treat odontogenic tumour and has a high recurrence rate. New evidence suggests that non-coding RNAs (ncRNAs) play a critical role in tumourgenesis and prognosis of cancer. However, ameloblastoma ncRNA expression data is lacking. Here we present the first report of ameloblastoma ncRNA signatures. A total of 95 ameloblastoma cases and a global array transcriptome technology covering > 285.000 full-length transcripts were used in this two-step analysis. The analysis first identified in a test cohort 31 upregulated ameloblastoma-associated ncRNAs accompanied by signalling pathways of cancer, spliceosome, mRNA surveillance and Wnt. Further validation in an independent cohort points out the long non-coding (lncRNAs) and small nucleolar RNA (snoRNAs): LINC340, SNORD116-25, SNORA11, SNORA21, SNORA47 and SNORA65 as a distinct ncRNA signature of ameloblastoma. Importantly, the presence of these ncRNAs was independent of BRAF-V600E and SMO-L412F mutations, histology type or tumour location, but was positively correlated with the tumour size. Taken together, this study shows a systematic investigation of ncRNA expression of ameloblastoma, and illuminates new diagnostic and therapeutic targets for this invasive odontogenic tumour.

## INTRODUCTION

Ameloblastoma is a benign but locally aggressive odontological neoplasm. The tumour is locally damaging to surrounding tissues, with great rate of recurrence and possibility to undergo malignant alteration. Majority of the tumours frequently develops in the mandible region and those in the maxilla are prone to spread into adjacent vital regions due to lack of cortical plates. Recent studies revealed that some ameloblastomas harbour BRAF and SMO mutations that may render them sensitive to new small molecule therapies. BRAF-V600E mutant ameloblastoma might be as frequent as up to 40%, and BRAF-targeted therapy has shown to rescue a patient suffering from malignant BRAF-V600E mutant ameloblastoma [1, 2]. Apart from this, the only approved treatment of choice today is surgery and jaw resection, which often causes severe morbidity and orofacial deformation and severe dysfunctions.

The identification of precise RNA transcripts or cluster of transcripts that are differentially expressed have led to an in-depth understanding of tumour biology as well as improvement of molecular diagnostics for cancer. RNA-based prognostic and therapeutic cancer markers have tremendous values, as a tumour RNA landscape determines the initiation, proliferation, and morphology of the tumour. Techniques like global transcriptome microarray and RNA-sequencing have been immense supports that have extended the view of tumour RNA landscape. In the genome of a living cell, protein-coding transcripts are the most well defined accounting for less than 2% of the entire genome [3, 4], whilst as much as 75% of the genome is actively transcribed into non-coding RNAs (ncRNAs) [5]. Despite that ncRNA transcripts do not code for proteins they are known to influence the different stages of gene expression from transcription and mRNA stability to mRNA translation. Emerging research suggests ncRNAs as new players in the cancer paradigm with potential role in tumour genesis and their possibility to serve as biomarkers has been proposed [6, 7]. Numerous tumour-associated ncRNAs are found to correlate to survival rate in diverse cancer types including, non-small cell lung cancer, gastric cancer and breast cancer [8–10].

One group of ncRNAs that are well characterized are the microRNAs (miRNAs; 19-25 approximately nucleotides long). This group of ncRNAs are reported to potentially contribute to the development of tumours in sense of acting as either oncogenes or as tumour suppressors [11]. Lately, high-throughput genomic technologies have revealed ubiquitous transcription of the human genome, raising the characterization of a new group of ncRNAs; long non-coding RNAs (lncRNAs) and small nucleolar RNA (snoRNAs). Nevertheless, the role of these ncRNAs as compared to miRNAs is largely undefined.

Defining the tumour-associated RNA landscape is highly important as sequestrated RNA has profound roles in tumourgenesis and may point out diagnostic and therapeutic markers/targets to improve current ameloblastoma treatments. In the context of ameloblastoma, a handful of microarray studies have demonstrated the protein-coding transcript profile and genes responsible for the tumourgenesis and progression [12, 13]. To our knowledge, the ncRNA profile in ameloblastoma has never been examined before. The focus of the present study was thus to investigate the involvement of ncRNAs in a well-defined ameloblastoma cohort to increase the knowledge of the RNAs contributing to and promoting tumourgenesis in these difficult-to-treat odontogenic tumour patients. Therefore we aimed to identify specific ncRNAs of ameloblastoma that could have potential to serve as future diagnostic markers.

## RESULTS

### Microarray data analysis on total tumour transcriptome

**Figure 1:**
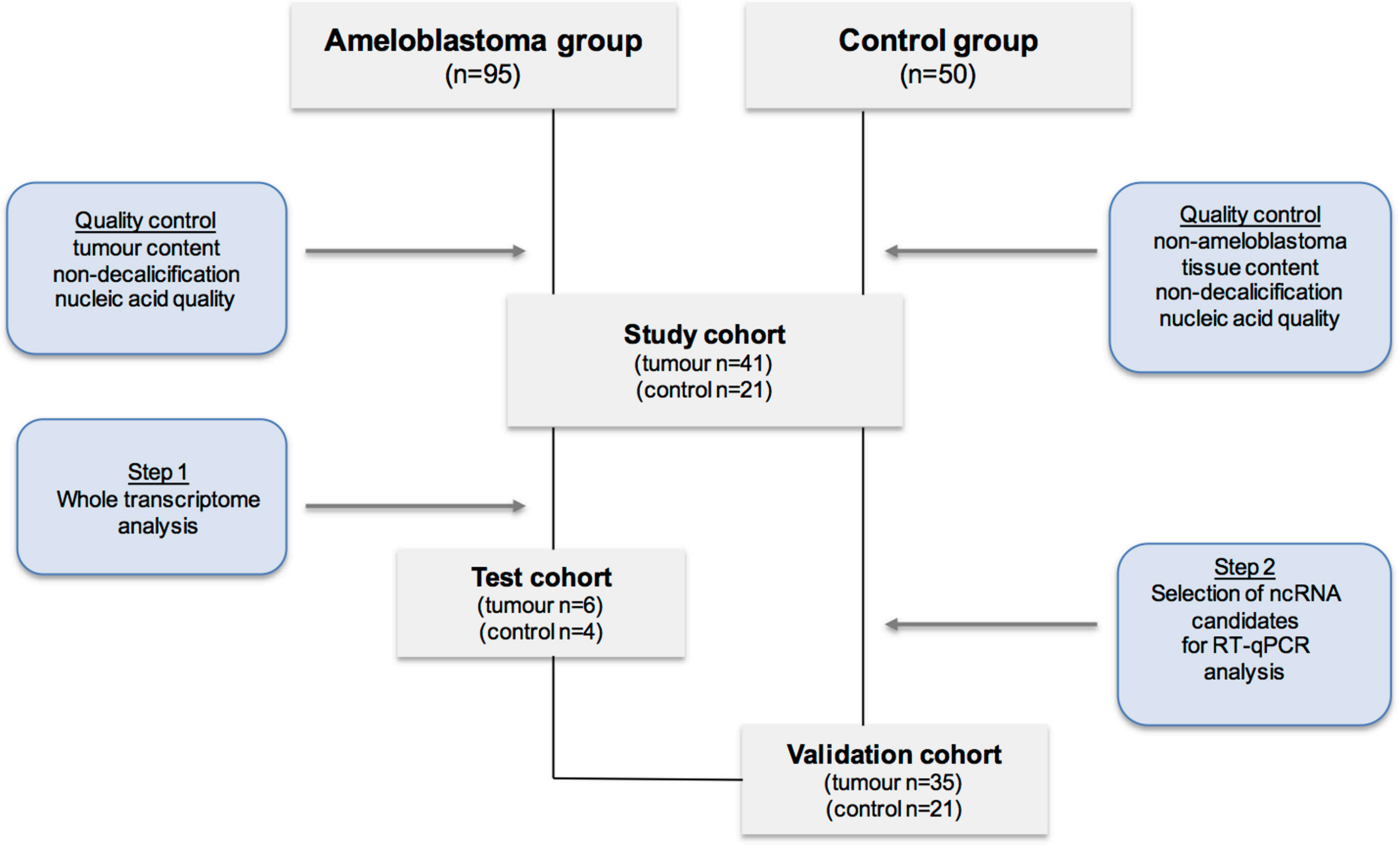
Flowchart describing the study design

**Figure 2:**
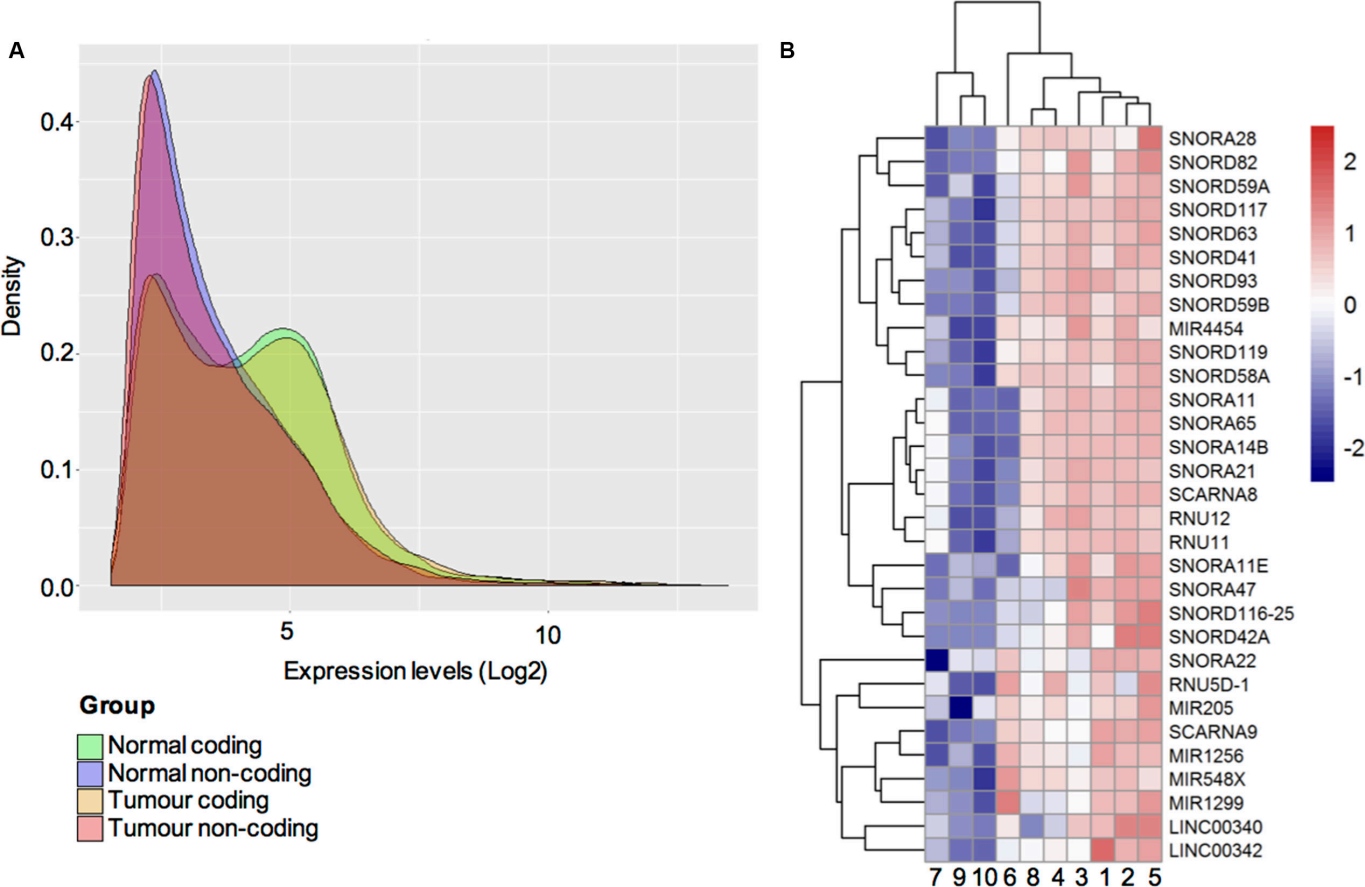
(**A**) Distribution of gene expression levels. A frequency histogram representing the distribution of expression levels of protein-coding and ncRNA transcripts in ameloblastoma tumours and non-ameloblastoma control tissues. The green peak indicates the expression of protein-coding genes in control tissue, orange peak distribution of coding genes in ameloblastoma, whereas pink peak indicates the expression of ncRNA in ameloblastoma tumours and purple peak the non-ameloblastoma control tissues. (**B**) Heat map and clustering dendrogram illustrating differential expression of ncRNAs in ameloblastoma and non-ameloblastoma tissue. In total 31 significantly upregulated ncRNAs were expressed in ameloblastoma tumour samples (samples: 1, 2, 3, 4, 5, 6) as compared to non-ameloblastoma control tissue (samples: 7: follicular cyst; 8: Oral lichen planus; 9–10: periradicular granuloma). The colour scale shown illustrates the relative expression level of ncRNAs across all samples. Colours can be interpreted using the scale where red colour represents an expression level > 2.0 in fold change and blue colour represents expression level <−2.0 in fold change. The length of the branch between two samples correspond to degree of similarity between the expressions of ncRNAs.

To examine the whole transcriptome including non-coding genes present in ameloblastoma tumours, we first chose to perform microarray analysis on a test cohort of histologically validated ameloblastoma (*n* = 6) and non-ameloblastoma oral tissue controls (*n* = 4). A flowchart describing the study design of this work is illustrated in Figure 1. The frequency histogram of gene levels from the microarray dataset in Figure 2A shows the distribution of expression levels of total protein-coding and non-coding transcripts detected in ameloblastoma tumours as well as non-ameloblastoma control tissues. Whilst differences in expression level between coding and non-coding transcripts detected were clear, the distribution of the detected transcripts was independent of tissue type (tumour or non-tumour).

Among the 245 000 human protein-coding transcripts and the 40 000 known ncRNA probes derived from RefSeq, Ensemble, IncRNAdb and Broad Institute, the microarray assay captured 85 significantly up-regulated (*p*-value < 0.05 and FC > 2.0) and 36 down-regulated (*p*-value < 0.05 and FC <−2.0) protein-coding transcripts in the ameloblastoma samples (Supplementary Table S1). The non-coding transcript analysis on the other hand revealed a total of 31 up-regulated non-coding transcripts - two lncRNAs, 24 snoRNAs, and five miRNAs in the ameloblastoma samples.

Identities of ameloblastoma-associated non-coding gene candidates (*p*-value < 0.05 and FC > 2.0) found here are summarised in Table 1, in which the chromosome location, transcript size, and their known involvement in other forms of neoplasms are indicated. The latter was determined in a meta-analysis where the specificity of the identified ncRNAs were investigated in public databases for potential correlations with other cancers. Of particular interest, the result (Table 1) revealed that LINC340, LINC342 and SNORD116-25 have been correlated to neuroblastoma, melanoma, small cell lung cancer, and bone marrow cells of multiple myeloma [14–16, 45]. SNORA65 overexpression in metastasised oral squamous cell carcinoma (OSCC) [17], SNORA21 and SNORA47 in lung cancer were also reported previously [8].

**Table 1:** Significantly up-regulated ncRNAs in ameloblastoma

Ensemble transcript ID	Gene symbol	Fold change tumour/control	Chromosome location	Transcript size	Involvement in other neoplasms
ENST00000516517	SNORD116-25	10.03	chr15	92	multiple myeloma [16]
ENST00000363299	RNU5D-1	5.06	chr1	116	**
ENST00000408789	SNORA11	5.00	chrX	128	**
ENST00000384262	SNORD63	4.47	chr5	68	**
ENST00000362512	RNU12	4.28	chr22	150	**
ENST00000515997	SNORD119	4.07	chr20	96	**
ENST00000362423	SNORA21	3.88	chr17	132	non-small cell lung cancer [8]
ENST00000515924	SCARNA8	3.87	chr9	131	**
ENST00000364432	SNORA65	3.85	chr9	130	Oral squamous cell carcinoma [17]
ENST00000458862	SNORA47	3.62	chr5	138	non-small cell lung cancer [8]
ENST00000386967	SNORD41	3.58	chr19	70	**
ENST00000530422	SCARNA9	3.51	chr11	353	**
ENST00000384452	SNORA14B	3.48	chr1	135	**
ENST00000364915	SNORD117	3.24	chr6	76	prostate cancer [44]
ENST00000580069	MIR548X	3.15	chr21	75	breast cancer [36]
ENST00000365530	SNORD82	3.02	chr2	70	**
ENST00000383875	SNORD58A	3.00	chr18	65	**
ENST00000408813	SNORD93	2.92	chr7	74	**
ENST00000408778	SNORA11E	2.86	chrX	128	**
ENST00000362779	SNORD59B	2.82	chr12	75	**
ENST00000622265	MIR1299	2.80	chr9	83	**
ENST00000387069	RNU11	2.72	chr1	134	**
ENST00000444265	LINC00340	2.59	chr6	1497	melanoma, neuroblastoma [14, 15]
ENST00000384304	SNORD59A	2.50	chr12	75	**
ENST00000384891	MIR205	2.49	chr12	110	head and neck squamous cell carcinoma, prostate, breast, glioblastoma, endometrial cancer [18, 30–33]
ENST00000412393	LINC00342	2.48	chr2	654	non-small cell lung cancer [45]
ENST00000383907	SNORA22	2.45	chr7	134	**
ENST00000408881	MIR1256	2.41	chr1	119	prostate cancer [38]
ENST00000606769	SNORA28	2.38	chr14	126	**
ENST00000459584	SNORD42A	2.31	chr17	64	**
---	MIR4454	2.30	chr4		bladder cancer [37]

Significant up-regulation were considered transcripts with a *p*-value < 0.05 and a fold change > 2.0

** No involvement reported in other neoplasm

### Clustering analysis on non-coding RNA identifies unique tumour-associated transcripts

The differential expression of the most significant ncRNAs (*p*-value < 0.05) in ameloblastoma as compared to non-ameloblastoma control tissues in this test-cohort were analysed and shown in Figure 2B. Here the relative expression level of ncRNAs across all samples (ameloblastoma and non-ameloblastoma controls) are illustrated in colour scale (red: FC > 2.0, blue FC < −2.0), the length of the branch between each sample correspond to the degree of similarity found between their ncRNA expression profiles. The cluster analysis showed that ameloblastomas (samples 1, 2, 3, 4, 5 and 6) clustered together sharing a similar pattern of ncRNAs expression. The non-ameloblastoma samples (7: follicular cyst; 8: oral lichen planus; 9–10: periradicular granuloma.) on the other hand clustered together, with exception of sample 8 the oral lichen planus which unlike the other jaw conditions is a precancerous mucosal disorder.

### Functional analysis of protein-coding gene composition confirms unique genetic pathway makeup of ameloblastoma

The biological functions of protein-coding genes associated with ameloblastoma in retrieved microarray data in a KEGG-pathway analysis further confirmed strong dominance of genes related to cancer pathways, spliceosome, basal cell carcinoma, mRNA surveillance pathway, Wnt and Notch signalling pathways, whereas calcium and genes involved in phosphorylation pathways were found to be down-regulated. The result is shown in Table 2 together with the number of regulated genes in respective pathway and the significance of enrichment.

**Table 2:** Enriched KEGG signalling pathways among up- and down-regulated protein-coding genes

Enriched KEGG signalling pathways	Genes in pathway (n)	*P*-value*
***Up-regulated pathways***		
Pathways in cancer	23	1.7 ×10^−6^
Spliceosome	13	1.4 ×10^−5^
Basal cell carcinoma	9	1.4 ×10^−5^
mRNA surveillance pathway	10	4.6 ×10^−5^
Wnt signalling pathway	12	0.0003
Notch signalling pathway	6	0.0036
Small cell lung cancer	7	0.0088
Prostate cancer	7	0.0088
Glioma	6	0.0088
Focal adhesion	11	0.0088
***Down-regulated pathways***		
Porphyrin and chlorophyll metabolism	5	0.0009
Renin-angiotensin system	3	0.0072
Neuroactive ligand-receptor interaction	8	0.0115
Cytokine-cytokine receptor interaction	8	0.0115
Pathogenic Escherichia coli infection	4	0.0115
Alzheimer's disease	6	0.0152
Calcium signalling pathway	6	0.0155
SNARE interactions in vesicular transport	3	0.0155
Metabolic pathways	18	0.0043
Oxidative phosphorylation	5	0.0168

* *P*-value indicates the significance of the enrichment (*p*-value < 0.05)

### LncRNA and snoRNA overexpression in validation cohort

**Figure 3:**
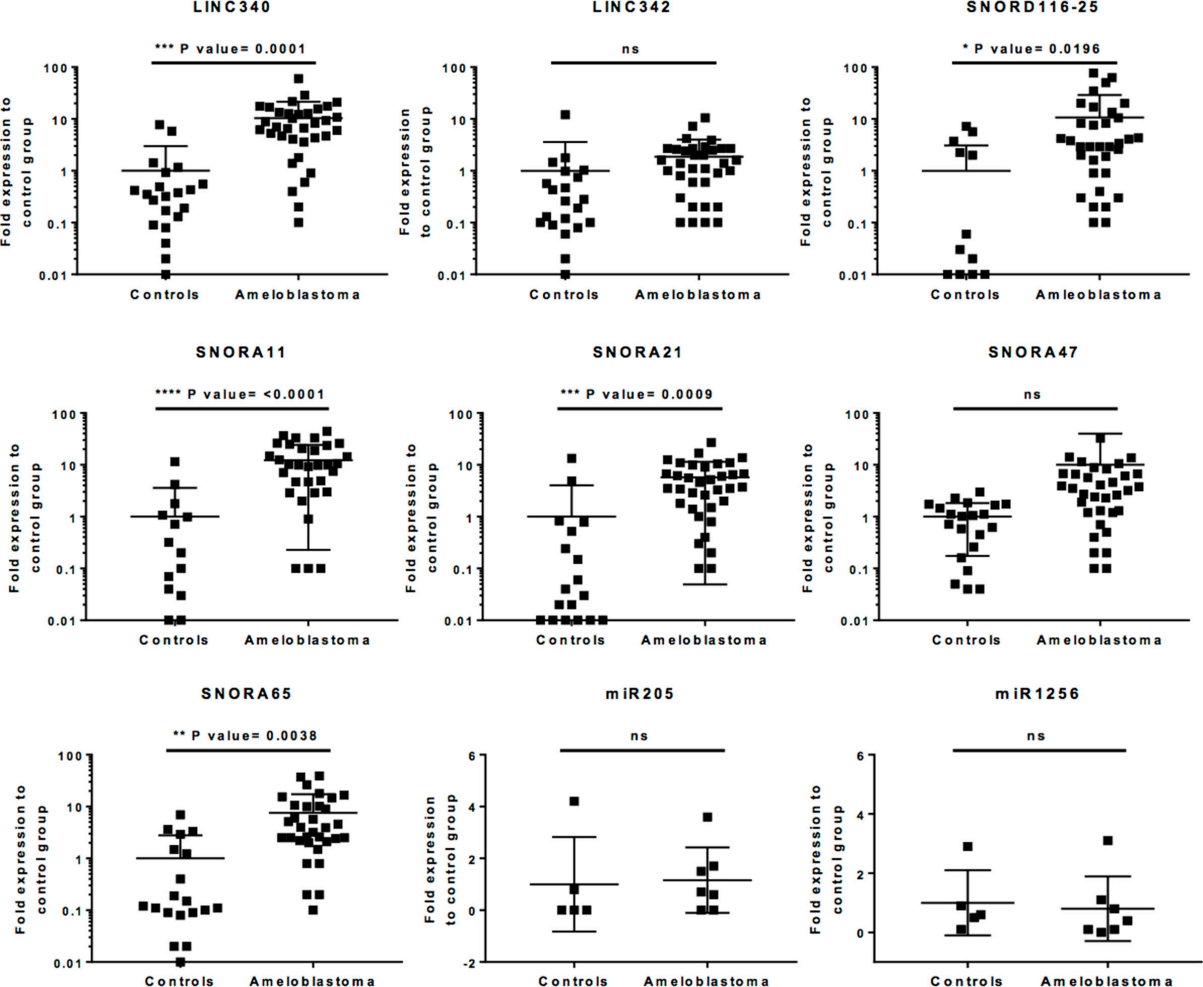
Uniquely expressed ncRNAs in ameloblastoma tumours Total RNA-extracted (DNAse treated) from 35 ameloblastoma samples and 21 non-ameloblastoma oral tissues were tested in qPCR using site-specific primers for each ncRNA candidate showing significantly increased relative expression in microarray or high cancer relevance. Dot plots show the individual ncRNA fold expression level of each tumour sample relative to the expression level of control group, including the group mean values, standard deviations and *p*-values. To normalise the ncRNA expression level, internal controls, GAPDH and RNU48 were used. Unpaired 2-tailed *t*-tests, was used for statistical comparison between the groups, statistical significance was defined as *p*-value < 0.05.

An independent validation cohort of histologically verified ameloblastoma tumour cases (*n* = 35) were used to further study the ncRNA candidates identified by microarray analysis. Non-ameloblastoma tissue samples (*n* = 21) were used as control. On the basis on the known ncRNA transcript length permitting RT-qPCR analysis, and the cancer relevance, nine ncRNA candidates were chosen, individual targeted RT-qPCR assay was designed and performed. To circumvent DNA carryover and contamination, all RNA samples were treated with DNAse prior to qPCR, and controls including water only and cDNA without RT-enzymes were included in the assays. The obtained results indicate that five of the ncRNAs identified in microarray assay, belonging to lncRNAs and snoRNAs classes, were abundantly expressed in this ameloblastoma validation cohort (LINC340: *p*-value < 0.0001; SNORD116-25: *p*-value = 0.0196; SNORA11: *p*-value <0.0001; SNORA21: *p*-value = 0.0009; and SNORA65: *p*-value = 0.0038; Figure 3). It was also noted that not only that some of the ncRNA expression were exceptionally high - LINC340, and SNORD116-25 were 40–50 fold overexpressed relative to non-ameloblastoma controls; they were overexpressed in the vast majority of ameloblastoma tumours tested (82–93%). Of note, miR205 - a suggested molecular marker for metastatic head and neck squamous cell carcinoma (HNSCC) [18] and miR1256 that were chosen for qPCR validation was found inconsistently expressed among ameloblastoma compared to non-ameloblastoma controls in this validation cohort (Figure 3).

**Figure 4:**
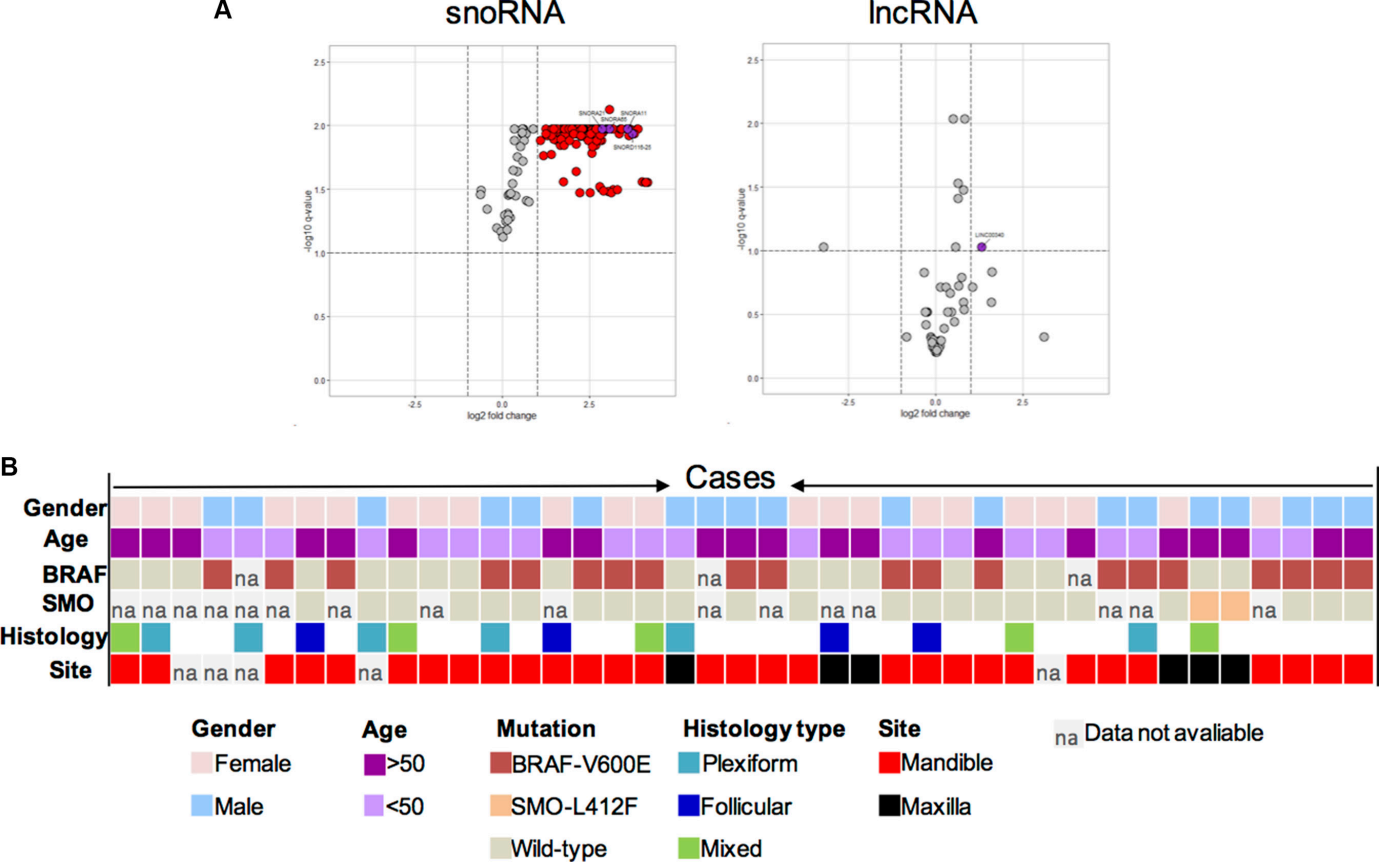
(**A**) Volcano plots of microarray data for snoRNA (A) and lincRNA (**B**) on microarray data. The data is plotted as log2 fold change versus the −log10 of the adjusted q-value. Thresholds are shown as dashed lines. ncRNAs that were significantly upregulated and confirmed in the validation cohort are shown as purple dots. ncRNAs that are significantly up-regulated are shown as red dots, as compared with non-tumour control tissues 7, 9, and 10. (B) Mutation frequency, distribution and relationship with pathological features. Information on age, gender, mutation status for the BRAF-V600E and SMO-L412F, histology as well as anatomical site is included below for each case, where each column represents a single case (in total 41). Colours correspond to a patient age, sex, and tumour mutation (BRAF-V600E; SMO-L412F), histology and anatomical site.

Differentially expressed non-coding RNAs in the microarray data were further visualised in volcano plots (Figure 4A and 4B) that illustrate the significance (e.g. FDR) versus fold-change for the gene microarray result. The result corroborates that SNORA65, SNORA21, SNORA47, SNORD116-25 and LINC340, which were validated here by RT and qPCR assays, belonged to the top most significantly expressed snoRNAs and lncRNAs in ameloblastoma.

### ncRNA sequestration is independent of BRAF and SMO mutations

Next the validated ncRNA candidates were correlated with the clinicopathological parameters of this validation cohort. The anatomical site, histological type of the tumour, sex and age of the patient, as well as BRAF-V600E and SMO-L412F mutations known to be common in ameloblastoma, were found to be independent of the level of ncRNA detected here. In accordance to earlier studies the BRAF-V600E mutation was frequent (20 out of 41 samples; 49%) in the tumour samples predominantly in the mandible region, whilst the SMO-L412F mutation was detected in two (5%) of the tumour samples only, exclusively in the maxilla region (Figure 4). No significant association between ncRNA and DNA point mutation type or clinicopathological features was observed (Figure 4).

### Association between ncRNAs fold expression and tumour size

**Figure 5:**
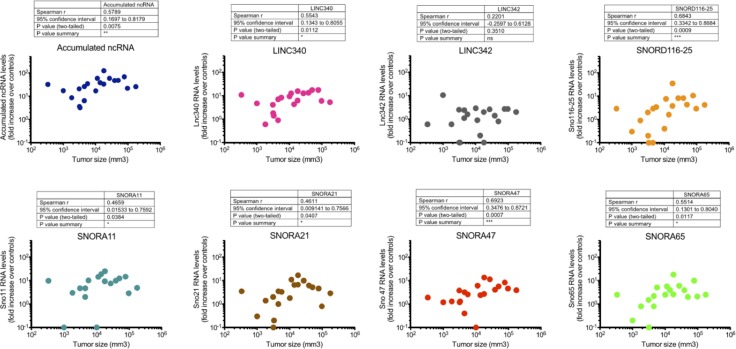
Association between ncRNAs fold expression and tumour size LINC340, SNORD116-25, SNORA11, SNORA21, SNORA47 and SNORA65 were significantly associated with the tumour size. Spearman non-parametric correlation test, two-tailed, 95% confidence interval was used for statistical comparison. Statistical significance was defined as *p*-value < 0.05.

Tumour size is considered an important determinant in clinical management of ameloblastoma. Of all cases examined here, 20 had complete radiographic documentation of tumour volume (mm^3^) that allowed correlation analysis with current findings. As shown in Figure 5, it was found that the accumulated ncRNA levels (LINC340, LINC342, SNORD116-25, SNORA11, SNORA21, SNORA47, SNORA65; r = 0.5789, *p*-value = 0.0075) had a positive but moderate correlation with tumour size. Interestingly, among these the SNORD116-25 (r = 0.6843, *p*-value = 0.0009) and SNORA47 (r = 0.6923, *p*-value = 0.0007) represent the most prominent correlates to tumour size whilst the other ones (except LINC342) were only weakly correlated to tumour size (Figure 5).

## DISCUSSION

Ameloblastoma remains the top difficult to treat odontogenic tumour and has a high recurrence rate. It is difficult to diagnose early, mainly due to the lack of screening imaging modalities and specific biomarkers. Slow growth initially and asymptomatic, consequently it is important to develop biomarkers that enable the detection of early stage tumours. Here we employed a global transcriptome approach to examine total ameloblastoma associated RNA in a two-step analysis; firstly, whole transcriptome profiling by microarray and secondly validation of of ncRNAs with RT and qPCR assays. Initial cluster analysis of the microarray data confirmed that ameloblastoma tumours shared a relative similar gene expression pattern different from the control tissues.

Our high-through-put first-step screening demonstrated coding and non-coding transcripts, the former confirmed ameloblastoma-specific protein encoding genes including parathyroid associated hormone-like proteins, amelotin and laminin [20–22], as well as functional pathway previously not reported but shared by other neoplasms including spliceosome, mRNA surveillance, Wnt and Notch signalling pathways. Supported by this result this analysis pointed out ncRNA candidates of interest to ameloblastoma, that belong to three major groups of ncRNAs (lncRNas, snoRNAs and miRNAs). The step two validation in 35 histologically validated tumour samples and relevant controls with qPCR led to conclusion that five unique ncRNAs – LINC340, SNORD116-25, SNORA11, SNORA21, and SNORA65 are highly associated to ameloblastoma.

Traditionally, immunohistochemical tumour biomarkers Ki-67, MCM2, and MCM3, CD138, p16 proteins that are known to be involved in cell proliferation have been used to elucidate histopathological features and clinical data of ameloblastoma Apart from Ki-67 that has been positively correlated with growth rate and shorter disease-free survival [23, 24] protein biomarkers have no significant association with clinical features of ameloblastoma [25]. In comparison to ncRNA, protein biomarkers require antibody capture and staining of sectioned biopsies. The features of ncRNA e.g. suitability for molecular detection and bioavailability in biofluids thus favour development of more sensitive PCR screenings on both puncture biopsies as well as liquid biopsies. The predictive ability of identified ncRNA thus extend beyond currently available protein markers, thus could be of use to monitor tumour growth and control this pathology to improve the quality of life of patients affected by this neoplasia.

LncRNAs are important regulators of gene expression, and thought to have a wide range of functions in cellular and developmental processes. Whereby, the number of lncRNAs involved in tumour genesis is rising rapidly. LncRNAs have been found to be differentially expressed in various types of cancer including leukemia breast cancer, hepatocellular carcinoma, colon cancer, and prostate cancer. Here we describe for the first time the identification and characterization of a novel lncRNA, LINC340, in the pathogenesis of ameloblastoma. LINC340 also known as Cancer Susceptibility Candidate 15 (CASC15) have likewise been implicated in other diseases including melanoma and neuroblastoma [14, 15]. One study reported that up-regulation of LINC340 is associated with melanoma progression to advance stages of distant metastasis. The study also suggested that LINC340 levels can be used to distinguish between patients at low and high risk of melanoma progression as well as an independent predictor of disease recurrence [15]. Furthermore, it has been reported that LINC340 is a mediator of neural growth and differentiation, which impacts neuroblastoma initiation and progression [14]. In this study we also found that the expression of LINC340 is positively correlated to the tumour lesion size suggesting a role of these ncRNA in tumour genesis of ameloblastoma.

snoRNA are ncRNAs that are not easily degraded in body fluids, making them attractive biomarkers. We have in this study reported significantly higher expression of a number of snoRNAs (SNORD116-25, SNORA11, SNORA21, SNORA47 and SNORA65) in ameloblastoma. Higher expression of SNORA21 and SNORA47 has earlier been associated to poor overall survival in patients with non-small cell lung cancer [8]. Furthermore, down-regulation of SNORA65 has been reported in chemo-sensitive ovarian adenocarcinoma compared to chemo-resistant tumours [26], and deregulation of SNORD116-25 in multiple myeloma [16]. In future studies it would be valuable to investigate the expression of these ncRNAs in biofluids in patients with ameloblastoma.

With regard to specificity, the ameloblastoma-associated ncRNAs found in present study was examined in a meta-analysis of aberrantly expressed ncRNA reported in other neoplasms. It was observed that aberrantly expressed LINC340 and SNORD116-25 have been found in melanoma, neuroblastoma, and multiple myeloma bone marrow cells [14–16]. SNORA11 has not until now been associated with other neoplasms, thus makes it unique in this perspective. Interestingly, SNORA65 has been correlated with metastasization in OSCC [17], SNORA21 and SNORA47 with lung cancer [8]. These notions are of great interest, as 1) OSCC is a common tumour of head and neck thus may have cell types that share tissue tropism with ameloblastoma; and 2) lungs are the most common site of metastatic ameloblastoma [1, 2]. To our knowledge a single sample data on ameloblastoma ncRNA has been embedded in a large human cancer dataset (GSM1631049) [27], though available in public database it does not allow a direct comparison to current data due to differences in study design.

Investigations across cancer types as Pan-Cancer Project recently led to identification of a panel of universal cancer biomarkers for 12 different types of cancers [28, 29]. These include 6 lincRNAs and 5 miRNAs - linc-Fam65c (PCAN-1), AL589743.1 (PCAN-2), LNCG006196 (PCAN-3), XLOC_l2_007509 (PCA-4) AP000525.9 (PCAN-5), XLOC_l2_013931 (PCAN-6), miR590, miR19a, miR93, miR200c and miR130b. Though some of them share same chromosomal locations (chr14, chr2, chr22) as those found in this ameloblastoma cohort, they were not detected by the methods used here. Other aberrantly expressed miRNAs known to be associated with head and neck cancers such as miR375, miR29 family, miR204, and miR99, miR196, miR21, miR31, miR29b-1, -2, miR370, miR126, miR337, miR30e, miR106b and miR654) [19] were not detected here either. Furthermore, unlike other gastrointestinal or head neck tumours known to have large amount of miRNAs the present ameloblastoma cohorts show only five upregulated miRNAs. The miRNAs that we found to be overexpressed by microarray in ameloblastoma tumours included miR1299, miR1256, miR205, miR4454 and miR548X. miR205 have been identified in a number of tumours including, prostate, breast, glioblastoma and endometrial cancer [30–33]. They can act either as tumour suppressors by inhibiting proliferation and invasion, or as an oncogene through enabling tumour initiation and proliferation depending on the specific tumour content and genes [34]. In one study it was demonstrated that overexpression of the miRNA, miR205 in KB oral cell lines induces tumour suppression by up-regulating the cytokine Interleukin-24, (IL-24; known to exhibit tumour-suppressing functions) directly by targeting its promoter binding site [35]. Nevertheless, in our study the expression of IL-24 was not significantly up-regulated in ameloblastoma (FC 1.25 *p*-value = 0.6), however further studies are called for to investigate the whither or not miR205 act as an angel or a devil in the pathogenesis of ameloblastoma. Furthermore, miR548 has been implicated in breast cancer, by acting as an anti-oncogenic regulator [36], miR454 to be overexpressed in bladder tumours and matched urine [37], and miR1256 to be involved in prostate cancer by inhibiting cell growth and invasion [38]. To our knowledge this is the first study to report the expression of miR1299 in ameloblastoma or tumours. Nonetheless, when we aimed at validating the miRNA expression in additional ameloblastoma cohort through RT-qPCR none of the miRNAs exhibited changes in the samples of validation cohort assessed by RT-qPCR. Though, future studies are of need to investigate the expression of them in a larger cohort as well as in biofluids from patients with ameloblastoma.

Apart from the notions above, the ncRNAs, SNORA11 and miR1299 are entirely new ncRNA markers that to our knowledge have not been reported to be associated to any other diseases including cancer. This renders them as well as the others excellent candidates as tumour markers for ameloblastoma. However their location within the tumour and subcellular location is currently beyond the scope of this study and thus remains to be addressed, as we have no information if they originate from ameloblastoma tumour cells or surrounding stroma or immune cells since the latter also may secrete ncRNA in response to dysfunctional cellular activities. The functional properties and base-pairing with other transcripts (SNORA116 has been associated with FOX2 regulator) also encourages more in-depth investigations.

A limitation of present study is lack of functional information of the ncRNA signatures identified here. As the primary aim of this present study was to perform a case-control study to affirm the ameloblastoma non-coding RNA profile and their clinical relevance, the functional aspects of these ncRNAs thus need to be investigated in a focused experimentally designed study. This is of importance considering that ncRNAs are known to function independently as well as in concert with each other. These findings above with regards to ncRNAs in ameloblastoma, is partly supported by analysis of the protein-coding genes in the ameloblastoma tumours. Through our KEGG signalling pathway enrichment analysis, results suggested that a substantial number of protein-coding transcripts were shared between ameloblastoma and other cancer types, in particularly the classical KEGG cancer pathways.

In conclusion, we have for the first time demonstrated the ncRNA signature in ameloblastoma. Although much work is required to clarify the release mechanism, origin of tumour-specific RNAs, their targeting protein-coding genes, and selectivity of carrier complexes, ameloblastoma associated ncRNAs are largely unexplored and might represent novel clinical biomarkers.

## MATERIALS AND METHODS

### Patient samples

Formalin fixed and paraffin embedded (FFPE) samples as well as freshly preserved samples in RNA Later were obtained at Karolinska University Hospital the Clinical Pathology Biobank, the Clinic of Oral Maxillofacial Surgery and the Clinic of Endodontic Surgery under permission of Ethical Review Board Stockholm and Karolinska Bio bank Board. Written informed consent was obtained from all patients. A total number of 95 tissue samples diagnosed with ameloblastoma were identified. After selection with following inclusion criteria: histopathological tumour validation, tumour cell content (> 40%), non-decalcification, case description, a total of 41 ameloblastoma tumour tissues were included into the study. As ameloblastoma irrelevant controls another 21 non-ameloblastoma tissue samples of oral and non-oral origin (follicular cysts, oral planus lichen, periradicular granulomas, healthy oral mucosa, pancreas, kidney, duodenum, and stomach) were included in the study. The study design is illustrated in Figure 1.

### DNA and RNA isolation

DNA and RNA from unstained FFPE 10μm sections were extracted with AllPrep DNA/RNA FFPE kit following the manufacturer's protocol (Qiagen) with Deparaffinization Solution (Qiagen). The AllPrep DNA/RNA mini kit (Qiagen) was used for RNA Later preserved tissues. Disruption and homogenization of the tissues was performed using Stainless Steel beads and the TissueLyser II from Qiagen. All samples were DNAse treated prior to downstream applications. A NanoDrop ND 1000 (Thermo Scientific) spectrophotometer was used for quantification and purity of the genomic DNA and RNA as evaluated by the absorption ratio at 260/280 nm.

### BRAF and SMO gene mutation analysis

For detection of BRAF-V600E and SMO-L412F mutations, Sanger sequencing and pyrosequencing were performed. PCR-amplified BRAF and SMO-gene products following M13-tailed primer amplification (Life technologies: BRAF forward primer 5′TGTAAAA CGACGGCCAGTGACATACTTATTGACTCTAAGAGG 3′, reverse primer 5′CAGGAAACAGCTATGAC|CTCTAG TAACTCAGCAGCATCTCA3′ and SMO forward primer 5′TGTAAAACGACGGCCAGTGATGGGGACT CTGTGAGTGG3′, reverse primer 5′CAGGAAACAGCT ATGACCTGTTGCCCAACTGGTCCT3′) were verified by gel electrophoresis and purified with the QIAquick PCR Purification kit (Qiagen) and thereafter Sanger sequencing (Eurofin Genomics). PCR reactions were performed in a 50 μl mixture containing 100 ng genomic DNA, 10X DreamTaq buffer with 20 mM MgCl_2_, 25 mM dNTPs, 0.5 μM of each primer and 5U DreamTaq DNA polymerase (Thermo Scientific). Reactions were run under following conditions: initial denaturation at 95°C for 15 min, 30 cycles at 94°C for 30 sec, 56°C for 30 sec, 72°C for 30 sec, and final extension cycle at 72°C for 7 min. BRAF pyrosequencing mutational analysis was performed according to the manufacturer's protocol for the PyroMark Q24 (Qiagen) and the use of PCR primers previously described for BRAF codon 600 [39]. Ten ng genomic DNA was used for each PCR reaction. The pyrosequencing analysis was performed as described [40]. Sequencing primer for BRAF codon 600 5´TGATTTTGGTCTAGCTACA3′.

### Total RNA microarray

For microarray analysis the high resolution Affymetrix GeneChip^®^ Human Transcriptome Array 2.0 (Affymetrix) was used. Compromised by 285.000 full-length transcripts including 245.000 protein-coding transcripts and 40.000 non-coding transcripts, the array provides coverage and accuracy to identify all known transcript isoforms produced by a gene. The microarray experiment were done with six ameloblastoma RNA samples and four non-ameloblastoma control RNA samples using the SensationPlus™ FFPE Amplification and WT Labeling Kit (Affymetrix). The SensationPlus™ kit was used to efficiently produce adequate target of whole-transcriptome amplification for complete gene expression profiling.

### Reverse Transcription (RT) and real-time quantitative PCR (qPCR)

To validate the expression of human ncRNAs (LINCC340, LINC342, SNORD116-25, SNORA11, SNORA21, SNORA47, and SNORA65), 250 ng of RNA was used to synthesize cDNA in 15 ul total reaction volume using IScript cDNA Synthesis kit (Biorad). 1 ul of cDNA, SsoAdvanced™ Universal SYBR*^®^* Green Supermix (Biorad), and gene specific primers were used for quantitative real time PCR (qPCR). A pilot experiment was performed to ensure that designed primers amplified specific genes. For the validation of the miRNA expression (miR205 and miR1256) stem loop qPCR method was performed as described [41]. In summary, 10 ng of RNA and TaqMan^®^ MicroRNA Reverse Transcription Kit containing reverse transcription stem loop primers (Applied Biosystems) were used to generate miRNA specific cDNA. 1.5 ul of cDNA, miRNA specific TaqMan small RNA assay containing TaqMan probe and primers (Applied Biosystems), and TaqMan^®^ Universal PCR Master Mix II, No UNG (Applied Biosystems) were used to prepare PCR reactions. qPCR was performed using 7500 Fast real time PCR system (Applied Biosystems). The amount of transcripts was determined as relative to internal reference genes GAPDH and RNU48 using the formula: 2^(CT of reference- CT of gene) as described [42]. The comparison analysis between groups (ameloblastoma vs. non-ameloblastoma) were performed using unpaired 2-tailed *t*-tests, statistical significance was defined as *p*-value < 0.05.

### Statistical analysis of microarray data

CEL files from the scanning were analysed in Affymetrix Expression Console v1.4.1 through Robust Multichip Analysis (RMA) algorithm, including background correction, probe set summarization, and quantile normalization. Expression levels in ameloblastoma tumours and control samples were compared using unpaired 2-tailed *t*-tests, and corresponding false discovery rates were estimated using the Bioconductor package *q*-value. To identify the significantly differentially expressed protein-coding and non-coding genes in ameloblastoma tumours, fold change (FC) of ameloblastoma tumours to non-ameloblastoma control tissues was compared. The genes were then filtered based on *p*-value and FC. Genes with *p*-value < 0.05 and FC > 2.0 were assumed to be significantly regulated (up- or down).

### Functional analysis of differentially expressed genes

The WEB-based Gene Set Analysis Toolkit version 3 (WebGestalt) [43] was used to find gene ontology (GO terms that are significantly enriched in selected sets of genes with different expression patterns in relation to the frequency of their occurrence in the set of all genes (*p*-value < 0.05). Furthermore, Kyoto Encyclopedia of Genes and Genomes (KEGG) enrichment pathway analysis was also performed with the parameters: ID type: entrez gene, Reference set: *Homo sapiens*, Statistical method: hypergeometric, multiple test adjustment: Benjamini & Hochberg (BH). Significance level was set to *p*-values < 0.05 and minimum number of two genes for each category.

### Data availability

The microarray data in this publication have been deposited in NCBI's Gene Expression Omnibus and are accessible through GEO Series accession number GSE90518. (https://www.ncbi.nlm.nih.gov/geo/query/acc.cgi?acc = GSE90518).

## SUPPLEMENTARY MATERIALS TABLE





## References

[1] Sweeney RT, McClary AC, Myers BR, Biscocho J, Neahring L, Kwei KA, Qu K, Gong X, Ng T, Jones CD, Varma S, Odegaard JI, Sugiyama T (2014). Identification of recurrent SMO and BRAF mutations in ameloblastomas. Nat Genet.

[2] Kaye FJ, Ivey AM, Drane WE, Mendenhall WM, Allan RW (2015). Clinical and radiographic response with combined BRAF-targeted therapy in stage 4 ameloblastoma. J Natl Cancer Inst.

[3] Stein LD (2004). Human genome: end of the beginning. Nature.

[4] Ponting CP, Belgard TG (2010). Transcribed dark matter: meaning or myth?. Hum Mol Genet.

[5] Iyer MK, Niknafs YS, Malik R, Singhal U, Sahu A, Hosono Y, Barrette TR, Prensner JR, Evans JR, Zhao S, Poliakov A, Cao X, Dhanasekaran SM (2015). The landscape of long noncoding RNAs in the human transcriptome. Nat Genet.

[6] Galasso M, Sana ME, Volinia S (2010). Non-coding RNAs: a key to future personalized molecular therapy?. Genome Med.

[7] Esteller M (2011). Non-coding RNAs in human disease. Nat Rev Genet.

[8] Gao L, Ma J, Mannoor K, Guarnera MA, Shetty A, Zhan M, Xing L, Stass SA, Jiang F (2015). Genome-wide small nucleolar RNA expression analysis of lung cancer by next-generation deep sequencing. Int J Cancer.

[9] Nasrollahzadeh-Khakiani M, Emadi-Baygi M, Schulz WA, Nikpour P (2016). Long noncoding RNAs in gastric cancer carcinogenesis and metastasis. Brief Funct Genomics.

[10] Yang F, Liu YH, Dong SY, Ma RM, Bhandari A, Zhang XH, Wang OC (2016). A novel long non-coding RNA FGF14-AS2 is correlated with progression and prognosis in breast cancer. Biochem Biophys Res Commun.

[11] Koturbash I, Zemp FJ, Pogribny I, Kovalchuk O (2011). Small molecules with big effects: the role of the microRNAome in cancer and carcinogenesis. Mutat Res.

[12] Carinci F, Palmieri A, Delaiti G, Rubini C, Fioroni M, Martinelli M, Pezzetti F, Scapoli L, Piattelli A (2004). Expression profiling of ameloblastic carcinoma. J Craniofac Surg.

[13] Heikinheimo K, Jee KJ, Niini T, Aalto Y, Happonen RP, Leivo I, Knuutila S (2002). Gene expression profiling of ameloblastoma and human tooth germ by means of a cDNA microarray. J Dent Res.

[14] Russell MR, Penikis A, Oldridge DA, Alvarez-Dominguez JR, McDaniel L, Diamond M, Padovan O, Raman P, Li Y, Wei JS, Zhang S, Gnanchandran J, Seeger R (2015). CASC15-S Is a Tumor Suppressor lncRNA at the 6p22 Neuroblastoma Susceptibility Locus. Cancer Res.

[15] Lessard L, Liu M, Marzese DM, Wang H, Chong K, Kawas N, Donovan NC, Kiyohara E, Hsu S, Nelson N, Izraely S, Sagi-Assif O, Witz IP (2015). The CASC15 Long Intergenic Noncoding RNA Locus Is Involved in Melanoma Progression and Phenotype Switching. J Invest Dermatol.

[16] Ronchetti D, Todoerti K, Tuana G, Agnelli L, Mosca L, Lionetti M, Fabris S, Colapietro P, Miozzo M, Ferrarini M, Tassone P, Neri A (2012). The expression pattern of small nucleolar and small Cajal body-specific RNAs characterizes distinct molecular subtypes of multiple myeloma. Blood Cancer J.

[17] Oliveira-Costa JP, Gigliola M, Macedo M, Carraro D, Soares F (2014). cDNA and lincRNA microarrays: identification of expression patterns related to metastatization in oral squamous cell carcinoma (1048.6). The FASEB Journal.

[18] Fletcher AM, Heaford AC, Trask DK (2008). Detection of metastatic head and neck squamous cell carcinoma using the relative expression of tissue-specific mir-205. Transl Oncol.

[19] Zou AE, Ku J, Honda TK, Yu V, Kuo SZ, Zheng H, Xuan Y, Saad MA, Hinton A, Brumund KT, Lin JH, Wang-Rodriguez J, Ongkeko WM (2015). Transcriptome sequencing uncovers novel long noncoding and small nucleolar RNAs dysregulated in head and neck squamous cell carcinoma. RNA.

[20] Lim J, Ahn H, Min S, Hong SD, Lee JI, Hong SP (2006). Oligonucleotide microarray analysis of ameloblastoma compared with dentigerous cyst. J Oral Pathol Med.

[21] Ren C, Diniz MG, Piazza C, Amm HM, Rollins DL, Rivera H, Devilliers P, Kestler DP, Waite PD, Mamaeva OA, Macdougall M (2011). Differential enamel and osteogenic gene expression profiles in odontogenic tumors. Cells Tissues Organs.

[22] Poomsawat S, Punyasingh J, Vejchapipat P (2007). Expression of basement membrane components in odontogenic tumors. Oral Surg Oral Med Oral Pathol Oral Radiol Endod.

[23] Otero D, Lourenco SQ, Ruiz-Avila I, Bravo M, Sousa T, de Faria PA, Gonzalez-Moles MA (2013). Expression of proliferative markers in ameloblastomas and malignant odontogenic tumors. Oral Dis.

[24] Migaldi M, Sartori G, Rossi G, Cittadini A, Sgambato A (2008). Tumor cell proliferation and microsatellite alterations in human ameloblastoma. Oral Oncol.

[25] Carreon-Burciaga RG, Gonzalez-Gonzalez R, Molina-Frechero N, Bologna-Molina R (2015). Immunoexpression of Ki-67, MCM2, and MCM3 in Ameloblastoma and Ameloblastic Carcinoma and Their Correlations with Clinical and Histopathological Patterns. Dis Markers.

[26] Choi CH, Choi JJ, Park YA, Lee YY, Song SY, Sung CO, Song T, Kim MK, Kim TJ, Lee JW, Kim HJ, Bae DS, Kim BG (2012). Identification of differentially expressed genes according to chemosensitivity in advanced ovarian serous adenocarcinomas: expression of GRIA2 predicts better survival. Br J Cancer.

[27] Balbin OA, Malik R, Dhanasekaran SM, Prensner JR, Cao X, Wu YM, Robinson D, Wang R, Chen G, Beer DG, Nesvizhskii AI, Chinnaiyan AM (2015). The landscape of antisense gene expression in human cancers. Genome Res.

[28] Ching T, Peplowska K, Huang S, Zhu X, Shen Y, Molnar J, Yu H, Tiirikainen M, Fogelgren B, Fan R, Garmire LX (2016). Pan-Cancer Analyses Reveal Long Intergenic Non-Coding RNAs Relevant to Tumor Diagnosis, Subtyping and Prognosis. EBioMedicine.

[29] Liu Z, Zhang J, Yuan X, Liu B, Liu Y, Li A, Zhang Y, Sun X, Tuo S (2015). Detecting pan-cancer conserved microRNA modules from microRNA expression profiles across multiple cancers. Mol Biosyst.

[30] Verdoodt B, Neid M, Vogt M, Kuhn V, Liffers ST, Palisaar RJ, Noldus J, Tannapfel A, Mirmohammadsadegh A (2013). MicroRNA-205, a novel regulator of the anti-apoptotic protein Bcl2, is downregulated in prostate cancer. Int J Oncol.

[31] Iorio MV, Casalini P, Piovan C, Di Leva G, Merlo A, Triulzi T, Menard S, Croce CM, Tagliabue E (2009). microRNA-205 regulates HER3 in human breast cancer. Cancer Res.

[32] Yue X, Wang P, Xu J, Zhu Y, Sun G, Pang Q, Tao R (2012). MicroRNA-205 functions as a tumor suppressor in human glioblastoma cells by targeting VEGF-A. Oncol Rep.

[33] Zhang G, Hou X, Li Y, Zhao M (2014). MiR-205 inhibits cell apoptosis by targeting phosphatase and tensin homolog deleted on chromosome ten in endometrial cancer Ishikawa cells. BMC Cancer.

[34] Qin AY, Zhang XW, Liu L, Yu JP, Li H, Wang SZ, Ren XB, Cao S (2013). MiR-205 in cancer: an angel or a devil?. Eur J Cell Biol.

[35] Kim JS, Yu SK, Lee MH, Park MG, Park E, Kim SG, Lee SY, Kim CS, Kim HJ, Chun HS, Chun SW, Kim DK (2013). MicroRNA-205 directly regulates the tumor suppressor, interleukin-24, in human KB oral cancer cells. Mol Cells.

[36] Shi Y, Qiu M, Wu Y, Hai L (2015). MiR-548-3p functions as an anti-oncogenic regulator in breast cancer. Biomed Pharmacother.

[37] Armstrong DA, Green BB, Seigne JD, Schned AR, Marsit CJ (2015). MicroRNA molecular profiling from matched tumor and bio-fluids in bladder cancer. Mol Cancer.

[38] Li Y, Kong D, Ahmad A, Bao B, Dyson G, Sarkar FH (2012). Epigenetic deregulation of miR-29a and miR-1256 by isoflavone contributes to the inhibition of prostate cancer cell growth and invasion. Epigenetics.

[39] Richman SD, Seymour MT, Chambers P, Elliott F, Daly CL, Meade AM, Taylor G, Barrett JH, Quirke P (2009). KRAS and BRAF mutations in advanced colorectal cancer are associated with poor prognosis but do not preclude benefit from oxaliplatin or irinotecan: results from the MRC FOCUS trial. J Clin Oncol.

[40] Sundstrom M, Edlund K, Lindell M, Glimelius B, Birgisson H, Micke P, Botling J (2010). KRAS analysis in colorectal carcinoma: analytical aspects of Pyrosequencing and allele-specific PCR in clinical practice. BMC Cancer.

[41] Chen C, Ridzon DA, Broomer AJ, Zhou Z, Lee DH, Nguyen JT, Barbisin M, Xu NL, Mahuvakar VR, Andersen MR, Lao KQ, Livak KJ, Guegler KJ (2005). Real-time quantification of microRNAs by stem-loop RT-PCR. Nucleic Acids Res.

[42] Larrea E, Riezu-Boj JI, Aldabe R, Guembe L, Echeverria I, Balasiddaiah A, Gastaminza P, Civeira MP, Sarobe P, Prieto J (2014). Dysregulation of interferon regulatory factors impairs the expression of immunostimulatory molecules in hepatitis C virus genotype 1-infected hepatocytes. Gut.

[43] Wang J, Duncan D, Shi Z, Zhang B (2013). WEB-based GEne SeT AnaLysis Toolkit (WebGestalt): update 2013. Nucleic Acids Res.

