# Supplementary material for: Ameloblastoma RNA profiling uncovers a distinct non-coding RNA signature
Source: Oncotarget. 2016 Dec 10;8(3):4530–42. doi: 10.18632/oncotarget.13889 (PMC5354851; doi:10.18632/oncotarget.13889)
Supplement: Supplementary file 1 [file oncotarget-08-4530-s001.pdf]

Ameloblastoma RNA profiling uncovers a distinct noncoding RNA signature

Supplementary Materials

Supplemenatry Table S1: Significantly up-and down-regulated protein-coding genes in ameloblastoma. See Supplementary_Table_S1
