# Supplementary material for: Ameloblastoma RNA profiling uncovers a distinct non-coding RNA signature
Source: Oncotarget. 2016 Dec 10;8(3):4530–42. doi: 10.18632/oncotarget.13889 (PMC5354851; doi:10.18632/oncotarget.13889)
Supplement: Supplementary file 2 [file oncotarget-08-4530-s002.docx]

| **UP-REGULATED GENES** |  |  |  |  |
| --- | --- | --- | --- | --- |
| **Ensembl ID** | **Gene symbol** | **Gene description** | **Fold change** | ***p*-value** |
| ENST00000201015 | PTHLH | parathyroid hormone-like hormone | 12,37 | 0,0417 |
| ENST00000296585 | ITGA2 | integrin, alpha 2 (CD49B, alpha 2 subunit of VLA-2 receptor) | 5,69 | 0,0396 |
| ENST00000222399 | LAMB1 | laminin, beta 1 | 4,98 | 0,0034 |
| ENST00000339336 | AMTN | amelotin | 4,49 | 0,0499 |
| ENST00000374694 | FZD8 | frizzled family receptor 8 | 4,37 | 0,0204 |
| ENST00000442267 | TBC1D9 | TBC1 domain family, member 9 (with GRAM domain) | 4,32 | 0,0271 |
| ENST00000245479 | SOX9 | SRY (sex determining region Y)-box 9 | 4,30 | 0,0007 |
| ENST00000310358 | SPON1 | spondin 1, extracellular matrix protein | 4,30 | 0,0415 |
| ENST00000498714 | FOXP1-IT1 | FOXP1 intronic transcript 1 (non-protein coding) | 4,07 | 0,0366 |
| ENST00000375856 | IRS2 | insulin receptor substrate 2 | 3,76 | 0,0029 |
| ENST00000377044 | TMEM2 | transmembrane protein 2 | 3,71 | 0,0417 |
| ENST00000339241 | SPRY1 | sprouty homolog 1, antagonist of FGF signaling (Drosophila) | 3,66 | 0,0110 |
| ENST00000264012 | CDH3 | cadherin 3, type 1, P-cadherin (placental) | 3,53 | 0,0310 |
| ENST00000279488 | DUSP6 | dual specificity phosphatase 6 | 3,50 | 0,0010 |
| ENST00000306732 | PITX2 | paired-like homeodomain 2 | 3,48 | 0,0402 |
| --- | ARL17A | ADP-ribosylation factor-like 17A | 3,44 | 0,0359 |
| ENST00000380666 | BNC2 | basonuclin 2 | 3,44 | 0,0293 |
| ENST00000282030 | SETBP1 | SET binding protein 1 | 3,27 | 0,0365 |
| ENST00000381271 | KLHL42 | kelch-like family member 42 | 3,22 | 0,0364 |
| ENST00000381273 | MANSC4 | MANSC domain containing 4 | 3,21 | 0,0487 |
| ENST00000256062 | TMTC1 | transmembrane and tetratricopeptide repeat containing 1 | 3,11 | 0,0179 |
| ENST00000239938 | EGR1 | early growth response 1 | 3,06 | 0,0199 |
| ENST00000260147 | FAT1 | FAT atypical cadherin 1 | 3,04 | 0,0078 |
| ENST00000260128 | SULF1 | sulfatase 1 | 3,00 | 0,0342 |
| ENST00000361566 | KRT19 | keratin 19 | 2,99 | 0,0029 |
| ENST00000266671 | PHLDA1 | pleckstrin homology-like domain, family A, member 1 | 2,98 | 0,0011 |
| ENST00000303562 | FOS | FBJ murine osteosarcoma viral oncogene homolog | 2,94 | 0,0094 |
| ENST00000240285 | RDH10 | retinol dehydrogenase 10 (all-trans) | 2,87 | 0,0119 |
| ENST00000296181 | ITGB5 | integrin, beta 5 | 2,85 | 0,0018 |
| ENST00000319349 | ETV4 | ets variant 4 | 2,73 | 0,0249 |
| ENST00000395925 | GLI3 | GLI family zinc finger 3 | 2,67 | 0,0112 |
| ENST00000361311 | CLSTN1 | calsyntenin 1 | 2,64 | 0,0039 |
| ENST00000342463 | SAT1 | spermidine/spermine N1-acetyltransferase 1 | 2,57 | 0,0036 |
| ENST00000289105 | ANKRD36 | ankyrin repeat domain 36 | 2,56 | 0,0233 |
| ENST00000199940 | MAP2 | microtubule-associated protein 2 | 2,55 | 0,0314 |
| ENST00000206514 | SLC7A8 | solute carrier family 7 (amino acid transporter light chain, L system), member 8 | 2,55 | 0,0473 |
| ENST00000318779 | FOXP1 | forkhead box P1 | 2,52 | 0,0130 |
| --- | PFN1P2 | profilin 1 pseudogene 2 | 2,52 | 0,0455 |
| ENST00000382020 | TUSC3 | tumor suppressor candidate 3 | 2,45 | 0,0270 |
| ENST00000240095 | SLC39A14 | solute carrier family 39 (zinc transporter), member 14 | 2,45 | 0,0092 |
| ENST00000329613 | TSHZ2 | teashirt zinc finger homeobox 2 | 2,43 | 0,0240 |
| ENST00000325006 | AHCYL2 | adenosylhomocysteinase-like 2 | 2,43 | 0,0268 |
| ENST00000293308 | KRT8 | keratin 8 | 2,42 | 0,0004 |
| ENST00000355029 | NET1 | neuroepithelial cell transforming 1 | 2,42 | 0,0375 |
| ENST00000357214 | SFPQ | splicing factor proline/glutamine-rich | 2,41 | 0,0072 |
| ENST00000358517 | PLEKHG1 | pleckstrin homology domain containing, family G (with RhoGef domain) member 1 | 2,41 | 0,0033 |
| ENST00000323874 | PTPRU | protein tyrosine phosphatase, receptor type, U | 2,40 | 0,0054 |
| ENST00000521091 | TUG1 | taurine upregulated 1 (non-protein coding) | 2,39 | 0,0116 |
| ENST00000330494 | CHD3 | chromodomain helicase DNA binding protein 3 | 2,39 | 0,0045 |
| ENST00000326648 | ZNF609 | zinc finger protein 609 | 2,38 | 0,0025 |
| ENST00000311852 | MMP14 | matrix metallopeptidase 14 (membrane-inserted) | 2,35 | 0,0279 |
| ENST00000407977 | RNF43 | ring finger protein 43 | 2,32 | 0,0025 |
| ENST00000339159 | ATP1A1 | ATPase, Na+/K+ transporting, alpha 1 polypeptide | 2,32 | 0,0055 |
| ENST00000443455 | ANKRD36B | ankyrin repeat domain 36B | 2,29 | 0,0226 |
| ENST00000372388 | DLG5 | discs, large homolog 5 (Drosophila) | 2,25 | 0,0007 |
| ENST00000368676 | ADAM12 | ADAM metallopeptidase domain 12 | 2,25 | 0,0454 |
| ENST00000265586 | ABCC5 | ATP-binding cassette, sub-family C (CFTR/MRP), member 5 | 2,24 | 0,0459 |
| --- | OR2A7 | olfactory receptor, family 2, subfamily A, member 7 | 2,23 | 0,0483 |
| ENST00000240304 | LUC7L3 | LUC7-like 3 (S. cerevisiae) | 2,23 | 0,0064 |
| ENST00000367021 | IRF6 | interferon regulatory factor 6 | 2,20 | 0,0163 |
| ENST00000307544 | KIAA1217 | KIAA1217 | 2,20 | 0,0154 |
| ENST00000252804 | PXDN | peroxidasin homolog (Drosophila) | 2,19 | 0,0421 |
| ENST00000256925 | CABLES1 | Cdk5 and Abl enzyme substrate 1 | 2,18 | 0,0063 |
| ENST00000286445 | GRIP1 | glutamate receptor interacting protein 1 | 2,17 | 0,0111 |
| ENST00000434704 | DLX3 | distal-less homeobox 3 | 2,17 | 0,0458 |
| ENST00000272849 | NRP2 | neuropilin 2 | 2,16 | 0,0137 |
| ENST00000375533 | BAMBI | BMP and activin membrane-bound inhibitor homolog (Xenopus laevis) | 2,16 | 0,0162 |
| ENST00000369239 | PNISR | PNN-interacting serine/arginine-rich protein | 2,15 | 0,0161 |
| ENST00000286201 | FZD7 | frizzled family receptor 7 | 2,15 | 0,0128 |
| ENST00000282441 | YAP1 | Yes-associated protein 1 | 2,15 | 0,0216 |
| ENST00000336749 | MOXD1 | monooxygenase, DBH-like 1 | 2,14 | 0,0193 |
| ENST00000377238 | PLXDC2 | plexin domain containing 2 | 2,12 | 0,0455 |
| ENST00000346192 | IGF2BP2 | insulin-like growth factor 2 mRNA binding protein 2 | 2,11 | 0,0027 |
| ENST00000458797 | SCARNA7 | small Cajal body-specific RNA 7 | 2,11 | 0,0031 |
| ENST00000330752 | HNRNPA1 | heterogeneous nuclear ribonucleoprotein A1 | 2,10 | 0,0440 |
| ENST00000313726 | ST5 | suppression of tumorigenicity 5 | 2,09 | 0,0100 |
| ENST00000260570 | IFT172 | intraflagellar transport 172 homolog (Chlamydomonas) | 2,08 | 0,0286 |
| ENST00000354858 | AK2 | adenylate kinase 2 | 2,04 | 0,0206 |
| ENST00000375926 | ARGLU1 | arginine and glutamate rich 1 | 2,04 | 0,0073 |
| ENST00000370489 | ENTPD7 | ectonucleoside triphosphate diphosphohydrolase 7 | 2,04 | 0,0010 |
| ENST00000221448 | SNRNP70 | small nuclear ribonucleoprotein 70kDa (U1) | 2,03 | 0,0160 |
| ENST00000277225 | ZNF462 | zinc finger protein 462 | 2,02 | 0,0095 |
| ENST00000513408 | GUSBP3 | glucuronidase, beta pseudogene 3 | 2,01 | 0,0154 |
| --- | SMA4 | glucuronidase, beta pseudogene | 2,01 | 0,0232 |
| ENST00000381633 | DDX17 | DEAD (Asp-Glu-Ala-Asp) box helicase 17 | 2,01 | 0,0138 |
| --- | ARL17B | ADP-ribosylation factor-like 17B | 2,00 | 0,0472 |
| **DOWN-REGULATED GENES** |  |  |  |  |
| **Ensembl ID** | **Gene symbol** | **Gene description** | **Fold change** | ***p*-value** |
| ENST00000252244 | KRT1 | keratin 1 | -55,47 | 0,0199 |
| ENST00000222032 | CNFN | cornifelin | -8,11 | 0,0216 |
| ENST00000341611 | SPRR2B | small proline-rich protein 2B | -7,81 | 0,0313 |
| ENST00000368750 | SPRR2E | small proline-rich protein 2E | -7,60 | 0,0300 |
| ENST00000274565 | SPINK7 | serine peptidase inhibitor, Kazal type 7 (putative) | -6,81 | 0,0264 |
| ENST00000444180 | FAM25A | family with sequence similarity 25, member A | -6,65 | 0,0197 |
| ENST00000381458 | TGM3 | transglutaminase 3 (E polypeptide, protein-glutamine-gamma-glutamyltransferase) | -6,06 | 0,0221 |
| ENST00000368790 | CRCT1 | cysteine-rich C-terminal 1 | -6,01 | 0,0337 |
| ENST00000264474 | CSTA | cystatin A (stefin A) | -5,98 | 0,0433 |
| ENST00000392653 | SPRR2A | small proline-rich protein 2A | -5,41 | 0,0197 |
| ENST00000340853 | FAM25C | family with sequence similarity 25, member C | -5,32 | 0,0137 |
| ENST00000246515 | SLURP1 | secreted LY6/PLAUR domain containing 1 | -4,54 | 0,0204 |
| ENST00000360379 | SPRR2D | small proline-rich protein 2D | -4,47 | 0,0199 |
| ENST00000307122 | SPRR1A | small proline-rich protein 1A | -4,40 | 0,0323 |
| ENST00000297991 | AQP3 | aquaporin 3 (Gill blood group) | -3,68 | 0,0321 |
| ENST00000339852 | NCCRP1 | non-specific cytotoxic cell receptor protein 1 homolog (zebrafish) | -3,52 | 0,0354 |
| ENST00000369705 | ME1 | malic enzyme 1, NADP(+)-dependent, cytosolic | -3,32 | 0,0147 |
| ENST00000368732 | S100A8 | S100 calcium binding protein A8 | -3,29 | 0,0498 |
| --- | SPRR2C | small proline-rich protein 2C (pseudogene) | -3,28 | 0,0262 |
| ENST00000368736 | S100A12 | S100 calcium binding protein A12 | -3,25 | 0,0323 |
| ENST00000325995 | KLHL38 | kelch-like family member 38 | -3,20 | 0,0250 |
| ENST00000345988 | PPL | periplakin | -3,04 | 0,0384 |
| ENST00000240617 | PLBD1 | phospholipase B domain containing 1 | -3,01 | 0,0113 |
| ENST00000375819 | LY6G6C | lymphocyte antigen 6 complex, locus G6C | -2,67 | 0,0381 |
| ENST00000272462 | MALL | mal, T-cell differentiation protein-like | -2,62 | 0,0314 |
| ENST00000388825 | GPX3 | glutathione peroxidase 3 (plasma) | -2,59 | 0,0248 |
| ENST00000380753 | AKR1C2 | aldo-keto reductase family 1, member C2 | -2,59 | 0,0437 |
| ENST00000343575 | CXCL12 | chemokine (C-X-C motif) ligand 12 | -2,55 | 0,0401 |
| ENST00000297373 | PHKG1 | phosphorylase kinase, gamma 1 (muscle) | -2,37 | 0,0482 |
| ENST00000260324 | SQRDL | sulfide quinone reductase-like (yeast) | -2,34 | 0,0262 |
| ENST00000320623 | NQO1 | NAD(P)H dehydrogenase, quinone 1 | -2,31 | 0,0145 |
| ENST00000368947 | ANXA9 | annexin A9 | -2,29 | 0,0142 |
| --- | NT5C1A | 5'-nucleotidase, cytosolic IA | -2,26 | 0,0202 |
| ENST00000355754 | GBP4 | guanylate binding protein 4 | -2,24 | 0,0114 |
| ENST00000201586 | SULT2B1 | sulfotransferase family, cytosolic, 2B, member 1 | -2,19 | 0,0500 |
| ENST00000359579 | AKR1B10 | aldo-keto reductase family 1, member B10 (aldose reductase) | -2,14 | 0,0330 |
| ENST00000268446 | MYLK4 | myosin light chain kinase family, member 4 | -2,04 | 0,0354 |
